# Updated racial disparities in incidence, clinicopathological features and prognosis of hypopharyngeal squamous carcinoma in the United States

**DOI:** 10.1371/journal.pone.0282603

**Published:** 2023-03-16

**Authors:** Zhong Liang, Meijuan Wu, Peng Wang, Huatao Quan, Jianqiang Zhao

**Affiliations:** 1 Head and Neck Surgery, The Cancer Hospital of the University of Chinese Academy of Sciences (Zhejiang Cancer Hospital), Institute of Basic Medicine and Cancer (IBMC), Chinese Academy of Sciences, Hangzhou, Zhejiang, China; 2 Department of Surgery, People’s Hospital of Haixi, Haixi Prefecture, Qinghai, China; 3 Department of Pathology, The Cancer Hospital of the University of Chinese Academy of Sciences (Zhejiang Cancer Hospital), The Key Laboratory of Zhejiang Province for Aptamers and Theranostics, Institute of Basic Medicine and Cancer (IBMC), Chinese Academy of Sciences, Hangzhou, Zhejiang, China; University of Auckland / University of Oxford, NEW ZEALAND

## Abstract

**Objective:**

This study was to determine the racial disparities in incidence, clinicopathological features and prognosis of hypopharyngeal squamous cell carcinoma (HPSCC) in the US.

**Methods:**

The National Program of Cancer Registries and Surveillance, Epidemiology, and End Results (SEER) database was used to determine racial disparity in age adjusted incidence rate (AAIR) of HPSCC and its temporal trend during 2004–2019. Using the separate SEER 17 database, we further evaluated racial disparity in clinicopathological features, and in prognosis using Kaplan-Meier curves and Cox proportional hazard models.

**Results:**

HPSCC accounted for 95.8% of all hypopharyngeal cancers and occurred much more frequently in males. Its incidence decreased in both male and females, in male non-Hispanic white (NHW), non-Hispanic black (NHB) and Hispanic as well as female NHW and NHB during the study period. NHB had the highest, whereas non-Hispanic Asian or Pacific Islanders (API) had comparable and the lowest incidence in both males and females. Among 6,172 HPSCC patients obtained from SEER 17 database, 80.6% were males and 83.9% were at the advanced stages III/IV. Five-year cancer specific and overall survival rates were 41.2% and 28.9%, respectively. NHB patients were more likely to be younger, unmarried, from the Southern region, larger sized tumor, and at the stage IV, but less likely to receive surgery. They also had higher proportions of dying from HPSCC and all causes. Multivariate analyses revealed that NHB with HPSCC at the locally advanced stage had both significantly worse cancer specific and overall survival compared with NHW, but not at early stage (I/II) or distant metastatic stage. Hispanic patients had significantly better prognosis than NHW at locally advanced and metastatic stages. NHW and API had comparable prognoses.

**Conclusions:**

HPSCC displays continuously decreased incidence and racial disparity. The majority of the disease is diagnosed at the advanced stage. NHB have the highest burden of HPSCC and a worse prognosis. More studies are needed to curtail racial disparity and improve early detection.

## Introduction

Hypopharyngeal cancer accounts for only approximately 3–6% of all head and neck malignancies [1, 2]. Its major histological subtype, squamous cell carcinoma, represents as high as 95% of all hypopharyngeal cancers [3]. Due to its anatomical features and asymptomatic presentation at an early stage, a high proportion of hypopharyngeal squamous-cell carcinoma (HSCC) is diagnosed in an advanced stage with a poor prognosis. Multidisciplinary treatments are required to improve locoregional control, preserve functions and enhance survival in HPSCC patients.

Due to disease rarity, very few studies have been conducted to investigate incidence and temporal change of hypopharyngeal cancers in recent years. An earlier study reported that the overall incidence of HPSCC was 1.0 per 100,000 persons in the US during 1973–2010. The incidence of HPSCC decreased with an average annual percent change (APC) of -2.0 (P<0.05). Its incidence reached 0.7 per 100,000 persons between 2006 and 2010 [4]. Another older study showed a similar decreased trend of HPSCC incidence during 1974–1999 [2]. No recent studies have updated HPSCC incidence and its temporal change in the US in recent years. Other studies have determined incidences of HPSCC in other countries [5, 6], or incidences of other head and neck cancers [7–9].

Several recent studies have built statistical models to predict the prognosis of HPSCC [10–16]. Race is found to be one of the independent factors correlated with survival in patients with HPSCC in some studies [10–13], in which black patients were shown to have worse survival compared to whites or other races [10–13, 17]. (However, other studies reported no racial disparity in the survival of patients with HPSCC [14–16]. Clinicopathological features of HPSCC among individual races have not been well investigated. Many of these studies divided races into blacks, whites and others without information for Hispanic, and Asian or Pacific Islanders. Using the database covering the entire population of the US, the objective of the present study was first to examine racial disparity of incidence of hypopharyngeal cancer and its temporal trend during 2004–2019. We then evaluated racial disparity in clinicopathological features and prognosis of HPSCC patients. Findings from this study may provide a public health interventional opportunity to address racial disparities and improve survival.

### Patients and methods

By utilizing the SEER*Stat 8.4.0, hypopharyngeal cancer patients diagnosed during 2004–2019 were extracted from the National Program of Cancer Registries (NPCR) and Surveillance, Epidemiology and End Results Program (SEER) incidence database [2001–2019] [18] which captures the entire population of the US. Hypopharyngeal cancer patients were selected based on the primary site ICD-O-3 codes of C12.9-C13.9. The histology of squamous cell carcinoma was based on the codes of 8050–8086 in histologic type ICD-O-3 [4]. The age adjusted incidence rates (AAIR) of hypopharyngeal cancer among different races were calculated by the SEER*Stat using the age-adjusted to the 2000 US standard population. Tiwari et al.’s 2006 modifications were applied for the confidence interval (CI) [19]. To avoid a variation in a single year, we also calculated the AAIR in 2017–2019 as the most current status of the disease. Races were divided into non-Hispanic white (NHW), non-Hispanic black (NHB), Hispanic, non-Hispanic Asian or Pacific Islander (API), non-Hispanic American Indians and Alaska Native (AIAN) and unknown.

Since the above NPCR-SEER incidence database has limited clinicopathological data and no individual information, the SEER Research Plus data 17 Registries [2000–2019] [20] were utilized to determine the impact of races and other clinicopathological factors on prognosis of HPSCC patients. Only patients diagnosed from 2004 to 2019 were included in this study. All patients were followed up to the end of the year 2019. We excluded patients with unknown survival time, T, N and M stage. AIAN and patients with unknown race were also excluded from analysis. Other variables included were age, sex, marital status, race, region, family income, tumor site, tumor size and treatment modality including surgery, radiotherapy, and chemotherapy. Based on the American Joint Committee on Cancer 8th Edition (AJCC) staging system, patients were categorized into early (stage I and II), locally advanced (III-IVb), and distant metastatic stage (IVc) [11, 15, 21]. Because SEER does not contain patient identity information, this study was exempt from review by the Hospital Ethic Committee.

### Statistical analysis

The temporal trend of AAIRs of hypopharyngeal cancers among races were analyzed by the Joinpoint Regression Program (version 4.9.0.1, DigitCompass LLC, Maryland, USA). Clinicopathological and prognostic data were analyzed using SAS software V9.4 (SAS Institute, Inc., Cary, NC.). The categorical data were examined using the χ^2^ test. Continuous data were analyzed by one-way ANOVA and the Tukey test for groupwise comparison. The Kaplan-Meier survival curves were generated and assessed by log-rank tests. Univariate and multivariate Cox proportional hazards models were used to determine the impact of clinicopathological factors on cancer-specific and overall survivals. The proportional hazard assumption was examined by including the time dependent covariates in the Cox model. All tests were two-sided and P values < 0.05 were considered to be statistically significant.

## Results

### Racial disparity in incidence rate of hypopharyngeal squamous cell carcinoma during 2004–2019

During the study period, there were a total of 37,076 subjects including 29,715 (80.1%) males and 7,361 (19.9%) females diagnosed with hypopharyngeal cancer (Table 1). Squamous cell carcinoma accounted for 96.0%, and 95.2% in males and females respectively. Among races, both Hispanic males (94.1%) and females (93.3%) had the lowest proportions of squamous cell carcinoma (Table 1). The overall AAIR for HPSCC during 2004–2019 was much higher in males (1.11 per 100,000 men) than in females (0.24 per 100,000 women). NHB had the highest AAIRs in both males and in females during the entire study period, and in most current years (2017–2019). Hispanic and API females had comparable and lowest AAIRs in both periods (Table 1).

**Table 1:** Accumulated case number and age adjusted incidence rate (AAIR) of hypopharyngeal squamous carcinoma (HPSCC) during 2004–2019.

Variable	Case number n (%)			AAIR (per 100,000)			
Period	2004–19			2004–19			2017–19
Histology	All	SCC	Others	All	SCC	Others	SCC
Overall	37,076	35,521 (95.8)	1,555 (4.2)	0.641	0.614	0.027	0.518
Male	29,715	28,514 (96.0)	1201 (4.0)	1.114	1.068	0.047	0.890
Female	7,361	7,007 (95.2)	1,201 (16.3)	0.240	0.228	0.012	0.200
Male							
NHW	22,000	21,136 (96.1)	864 (3.9)	1.077	1.033	0.043	0.886
NHB	4,758	4,578 (96.2)	180 (3.8)	1.849	1.772	0.077	1.272
Hispanic	1,914	1,806 (94.4)	108 (5.6)	0.867	0.817	0.050	0.692
API	719	694 (96.5)	25 (3.5)	0.691	0.664	0.028	0.661
Female							
NHW	5,768	5,482 (95.0)	286 (5.0)	0.251	0.238	0.012	0.211
NHB	1,023	990 (96.8)	33 (3.2)	0.307	0.297	0.010	0.242
Hispanic	326	305 (93.6)	21 (6.4)	0.118	0.110	0.008	0.108
API	150	142 (94.7)	8 (5.3)	0.108	0.102	0.006	0.112

API, Asian or Pacific Islander; NHB, non-Hispanic black; NHW, non-Hispanic white; SCC, squamous cell carcinoma.

**Fig 1:**
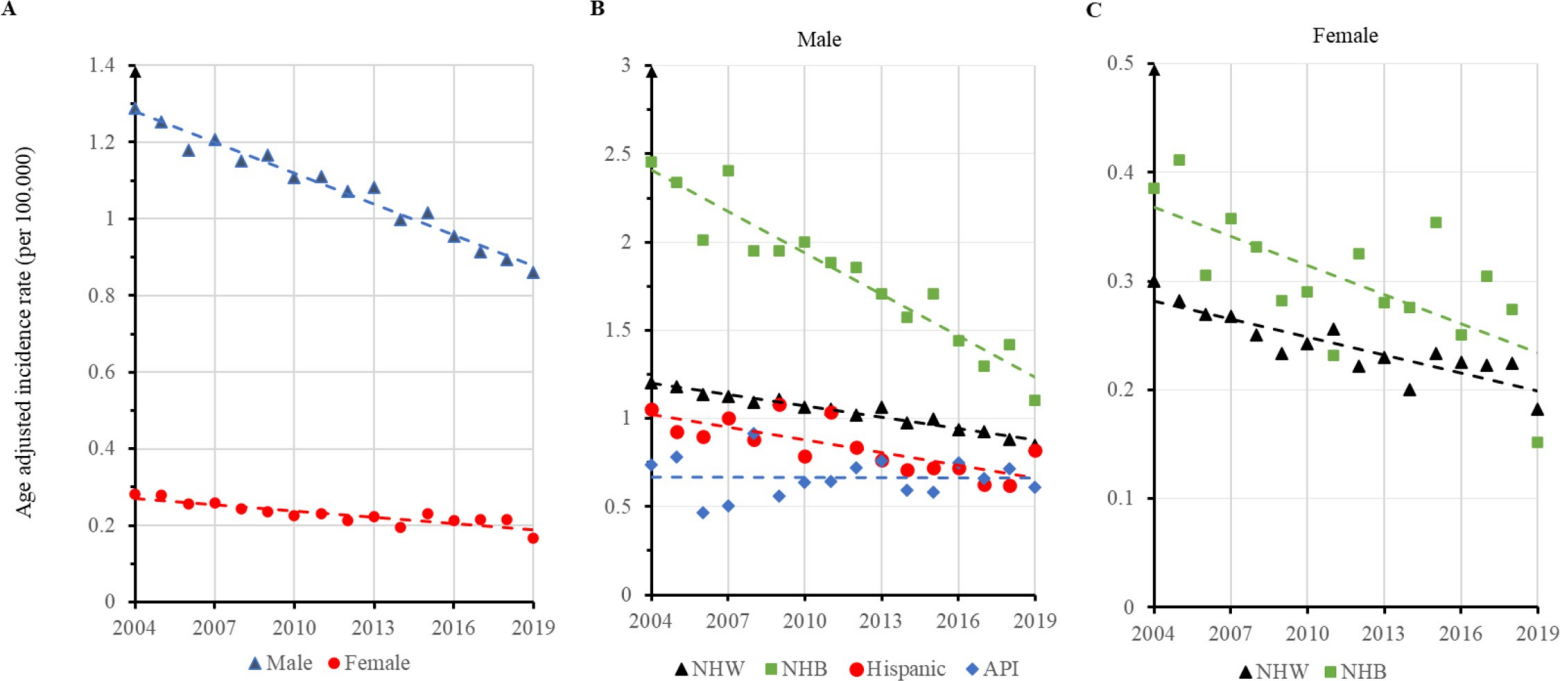
Age adjusted incidence rate (AAIR) of hypopharyngeal squamous cell carcinoma (HPSCC) by sex and race during 2004–2009. (**A**). Temporal change of overall AAIR in males and females. (**B**). Trends of AAIR in male non-Hispanic white (NHW), non-Hispanic black (NHB), Hispanic and Asian or Pacific Islanders (API). The lines indicate temporal trends. Data of female Hispanic and API were not available in many individual years and not presented.

Temporally, there were decreased trends of overall AAIR for HPSCC in both males and females of all races/ethnicities (Fig 1A). The APCs were -2.48 (95% confidence interval (CI): -.2.76 to -2.20, P<0.001) in males and -2.28 (95% CI: -.3.00 to -1.55, P<0.001) in females. Among individual races, the incidence rate was significantly decreased from 1.20 per 100,000 in 2004 to 0.85 in male NHW in 2019, with an APC of -2.06 (95% CI: -2.34 to -1.77, P<0.001) (Fig 1B). The AAIR in male NHB had the fastest rate of decrease with an APC of -4.30 (95% CI: -5.14 to -3.45, P<0.001), and from an AAIR of 2.46 in 2004 to 1.11 in 2019. Hispanic males displayed a decreased trend with an APC of -2.81 (95% CI: -4.20 to -1.39, P = 0.001). There was no significant change in the incidence rate in male API during the period (Fig 1B). Among females, AAIR of HPSCC showed significant decreases from 0.30 per 100,000 women to 0.18 with an APC of -2.25 (95% CI: -2.95 to to1.54, P<0.001) in NHW, and from 0.39 in 2004 to 0.15 in 2019 with an APC of -2.73 (95% CI: -4.65 to -0.78, P = 0.010) in NHB during the study period (Fig 1C). Data on HPSCC incidence rates in male and female AIANs, and female Hispanic and API in many years were not available in the database, and thus not presented.

### Characteristics of hypopharyngeal squamous carcinoma patients diagnosed during 2004–2019

After excluding patients with unknown information of survival time, and T, N, and M stages, 6172 patients with HPSCC were selected for further analysis from the SEER 17 database which covers 26.5% of the population in the US. Our data showed that over a half of the patients (50.1%) were diagnosed when they were 65 years or older, with the majority being males (80.6%) (Table 2). Among these patients, there were 70.6% NHW, 15.0% NHB, 8.2% Hispanic and 6.2% API. Around half of tumors was found at the pyriform sinus. Only 20.2% patients underwent surgery, whereas 77.4% patients received radiotherapy and 63.1% were treated with chemotherapy. A total of 3,299 (53.5%) patients died of this disease, and 4,592 (74.4%) patients died due to all causes (Table 2).

**Table 2:** Clinicopathological characteristics of patients with HPSCC by stage diagnosed during 2004–2019.

Variable	Overall	Early stage (I/II)	Locally advanced stage (III-IVb)	Distant metastasis (IVc)	P value
	n (%)	n (%)	n (%)	n (%)	
	6,172 (100)	991 (16.1)	4,679 (75.8)	502 (8.1)	
Age (years)					
Mean ± SD	65.2 ± 10.8	67.5 ± 11.1	64.7 ± 10.7	64.7 ± 10.4	< 0.0001
Median (range)	65 (20–98)	67 (31–98)	64 (20–97)	64 (39–93)	
≤ 65	3,078 (49.9)	406 (41.0)	2,408 (51.5)	264 (52.6)	< 0.0001
> 65	3,094 (50.1)	585 (59.0)	2,271 (48.5)	238 (47.4)	
Gender					<0.0001
Male	4,973 (80.6)	733 (74.0)	3,819 (81.6)	421 (83.9)	
Female	1,199 (19.4)	258 (26.0)	860 (18.4)	81 (16.1)	
Race					
NHW	4356 (70.6)	745 (75.2)	3,285 (70.2)	326 (64.9)	0.0004
NHB	926 (15.0)	120 (12.1)	705 (15.1)	101 (20.1)	
Hispanic	507 (8.2)	68 (6.9)	390 (8.3)	49 (9.8)	
API	383 (6.2)	58 (5.9)	299 (6.4)	26 (5.2)	
Marital status					
Married	2,855 (46.3)	539 (54.4)	2,114 (45.2)	202 (40.2)	<0.0001
Unmarried^#^	3,076 (49.8)	419 (42.3)	2,373 (50.7)	284 (56.6)	
Unknown	241 (3.9)	33 (3.3)	192 (4.1)	16 (3.2)	
Year of diagnosis					
2004–2009	2,284 (37)	373 (37.6)	1751 (37.4)	160 (31.9)	0.1434
2010–2014	2,086 (33.8)	336 (33.9)	1,573 (33.6)	177 (35.3)	
2015–2019	1,802 (29.2)	282 (28.5)	1,355 (29.0)	165 (32.9)	
Annual household income ($)					
≤ 50000	1,138 (18.4)	166 (16.8)	870 (18.6)	102 (20.3)	0.0174
50001–75000	3,336 (54.1)	582 (58.7)	2,485 (53.1)	269 (53.6)	
>75000	1,698 (27.5)	243 (24.5)	1,324 (28.3)	131 (26.1)	
Region					
West	2,893 (46.9)	456 (46.0)	2,221 (47.5)	216 (43.0)	0.0819
South	1,969 (31.9)	322 (32.5)	1,470 (31.4)	177 (35.3)	
Midwest	265 (4.3)	56 (5.7)	192 (4.1)	17 (3.4)	
Northeast	1,045 (16.9)	157 (15.8)	796 (17.0)	92 (18.3)	
Site					
Pyriform sinus	3,114 (50.5)	468 (47.2)	2,411 (51.5)	235 (46.8)	<0.0001
Postcricoid region	183 (3.0)	35 (3.5)	134 (2.9)	14 (2.8)	
Aryepiglottic fold	410 (6.6)	124 (12.5)	258 (5.5)	28 (5.6)	
Posterior wall	446 (7.2)	106 (10.7)	312 (6.7)	28 (5.6)	
Overlapping lesion	235 (3.8)	32 (3.2)	184 (3.9)	19 (3.8)	
Hypopharynx, NOS	1,784 (28.9)	226 (22.8)	1,380 (29.5)	178 (35.5)	
Size (cm)					
≤ 2	1,094 (17.7)	393 (39.7)	646 (13.8)	55 (11.0)	<0.0001
2.1–5	2,787 (45.2)	412 (41.6)	2,173 (46.4)	202 (40.2)	
>5	739 (12.0)	0 (0)	656 (14.0)	83 (16.5)	
Unknown	1,552 (25.2)	186 (18.8)	1,204 (25.7)	162 (32.3)	
T stage					
T1	668 (10.8)	289 (29.2)	355 (7.6)	24 (4.8)	<0.0001
T2	2,156 (34.9)	702 (70.8)	1,310 (28.0)	144 (28.7)	
T3	1,225 (19.9)	0 (0)	1,132 (24.2)	93 (18.5)	
T4	2,123 (34.4)	0 (0)	1,882 (40.2)	241 (48.0)	
N stage					
N0	1,875 (30.4)	991 (100)	826 (17.7)	58 (11.6)	<0.0001
N1	1,262 (20.5)	0 (0)	1,157 (24.7)	105 (20.9)	
N2	2,687 (43.5)	0 (0)	2,405 (51.4)	282 (56.2)	
N3	348 (5.6)	0 (0)	291 (6.2)	57 (11.4)	
M stage					
M0	5,670 (91.9)	991 (100)	4,679 (100)	0 (0)	<0.0001
M1	502 (8.1)	0 (0)	0 (0)	502 (100)	
Stage					
I	289 (4.7)	289 (29.2)	0 (0)	0 (0)	<0.0001
II	702 (11.4)	702 (70.8)	0 (0)	0 (0)	
III	1,112 (18.0)	0 (0)	1,112 (23.8)	0 (0)	
IV	4,069 (65.9)	0 (0)	3,567 (76.2)	502 (100)	
Grade					
I	262 (4.2)	64 (6.5)	183 (3.9)	15 (3.0)	<0.0001
II	2,541 (41.2)	473 (47.7)	1,894 (40.5)	174 (34.7)	
III/IV	1,919 (31.1)	264 (26.6)	1,494 (31.9)	161 (32.1)	
Unknown	1,450 (23.5)	190 (19.2)	1,108 (23.7)	152 (30.3)	
Dissected lymph node number					
0	4,189 (67.9)	873 (88.1)	2,959 (63.2)	357 (71.1)	
1–10	304 (4.9)	31 (3.1)	251 (5.4)	22 (4.4)	<0.0001
11–20	165 (2.7)	28 (2.8)	133 (2.8)	4 (0.8)	
>20	573 (9.3)	43 (4.3)	522 (11.2)	8 (1.6)	
Unknown	941 (15.3)	16 (1.6)	814 (17.4)	111 (22.1)	
Surgery					
No	4,924 (79.8)	693 (69.9)	3,761 (80.4)	470 (93.6)	<0.0001
Yes	1,248 (20.2)	298 (30.1)	918 (19.6)	32 (6.4)	
Chemotherapy					
No	2,279 (36.9)	646 (65.2)	1,450 (31)	183 (36.5)	<0.0001
Yes	3,893 (63.1)	345 (34.8)	3,229 (69)	319 (63.6)	
Radiotherapy					
No	1,394 (22.6)	295 (29.8)	893 (19.1)	206 (41.0)	<0.0001
Yes	4,778 (77.4)	696 (70.2)	3,786 (80.9)	296 (59.0)	
Cancer specific death					
No	2,873 (46.6)	613 (61.9)	2153 (46.0)	107 (21.3)	<0.0001
Yes	3,299 (53.5)	378 (38.1)	2,526 (54.0)	395 (78.7)	
Overall death					
No	1,580 (25.6)	329 (33.2)	1,208 (25.8)	43 (8.6)	<0.0001
Yes	4,592 (74.4)	662 (66.8)	3,471 (74.2)	459 (91.4)	

API, Asian or Pacific Islander; NHB, non-Hispanic black; NHW, non-Hispanic white; SCC, squamous cell carcinoma.

^#^ Includes divorces, separated, widowed and unmarried

We then assessed clinicopathological features at varied disease stages. Patients with HPSCC at the locally advanced stage (III-IVb), and distant metastatic (IVc) stage were significantly younger, with a higher proportion being males, unmarried, either NHB or Hispanic, tumor size >5 cm and grade III/IV (poorly or undifferentiated). They were more likely to receive chemotherapy, but less likely to undergo surgery. Patients with locally advanced HPSCC were more likely to receive radiotherapy compared with patients in early stages (I/II) (Table 2).

### Racial disparity in clinicopathological features of hypopharyngeal squamous carcinoma

NHB patients were significantly younger than other races/ethnicities (Table 3). They were more likely to be unmarried, lower income, from the Southern region, tumor size of > 5 cm, and IV stage. In contrast, NHB were the least to receive surgery, but a similar proportion to receive chemotherapy and radiotherapy. NHB also had significantly higher proportions of cancer specific death (59.6%) and overall death (80.7%) compared to other races (Table 3).

**Table 3:** Racial disparity of clinicopathological features in HPSCC patients diagnosed during 2004–2015.

Variable	NHW	NHB	Hispanic	API	P value
	n (%)	n (%)	n (%)	n (%)	
	4,356 (70.6)	926 (15.0)	507 (8.2)	383 (6.2)	
Age (years)					
Mean ± SD	65.7 ± 10.7	61.9 ± 9.9	64.4 ±11.5	67.7 ± 12.1	<0.0001
Median (range)	65 (26–98)	61 (23–91)	63 (20–94)	68 (32–95)	
≤ 65	2,065 (47.4)	586 (63.3)	275 (54.2)	152 (39.7)	
> 65	2,291 (52.6)	340 (36.7)	232 (45.8)	231 (60.3)	
Gender					0.0054
Male	3465 (79.6)	758 (81.9)	433 (85.4)	317 (82.8)	
Female	891 (20.5)	168 (18.1)	74 (14.6)	66 (17.2)	
Marital status					<0.0001
Married	2,116 (48.6)	261 (28.2)	229 (45.2)	249 (65.0)	
Unmarried^#^	2,074 (47.6)	625 (67.5)	254 (50.1)	123 (32.1)	
Unknown	166 (3.8)	40 (4.3)	24 (4.7)	11 (2.9)	
Year of diagnosis					<0.0001
2004–2009	1,652 (37.9)	366 (39.5)	152 (30.0)	114 (29.8)	
2010–2014	1,477 (33.9)	296 (32)	190 (37.5)	123 (32.1)	
2015–2019	1,227 (28.2)	264 (28.5)	165 (32.5)	146 (38.1)	
Annual household income ($)					<0.0001
≤ 50,000	1,652 (37.9)	366 (39.5)	152 (30.0)	114 (29.8)	
50,001–75,000	1,477 (33.9)	296 (32.0)	190 (37.5)	123 (32.1)	
>75,000	1,227 (28.2)	264 (28.5)	165 (32.5)	146 (38.1)	
Region					<0.0001
West	1,954 (44.9)	205 (22.1)	405 (79.9)	329 (85.9)	
South	1,365 (31.3)	559 (60.4)	25 (4.9)	20 (5.2)	
Midwest	254 (5.8)	6 (0.7)	2 (0.4)	3 (0.8)	
Northeast	783 (18.0)	156 (16.9)	75 (14.8)	31 (8.1)	
Site					0.0003
Pyriform sinus	2,236 (51.3)	420 (45.4)	246 (48.5)	212 (55.4)	
Postcricoid region	124 (2.9)	30 (3.2)	18 (3.6)	11 (2.9)	
Aryepiglottic fold	309 (7.1)	59 (6.4)	26 (5.1)	16 (4.2)	
Posterior wall	312 (7.2)	76 (8.2)	28 (5.5)	30 (7.8)	
Overlapping lesion	159 (3.7)	34 (3.7)	34 (6.7)	8 (2.1)	
Hypopharynx, NOS	1,216 (27.9)	307 (33.2)	155 (30.6)	106 (27.7)	
Size (cm)					
≤ 2	829 (19.0)	120 (13.0)	76 (15.0)	69 (18.0)	<0.0001
2.1–5	1,982 (45.5)	387 (41.8)	232 (45.8)	186 (48.6)	
>5	477 (11.0)	143 (15.4)	63 (12.4)	56 (14.6)	
Unknown	1,068 (24.5)	276 (29.8)	136 (26.8)	72 (18.8)	
T stage					<0.0001
T1	527 (12.1)	63 (6.8)	39 (7.7)	39 (10.2)	
T2	1,570 (36.0)	284 (30.7)	160 (31.6)	142 (37.1)	
T3	890 (20.4)	158 (17.1)	97 (19.1)	80 (20.9)	
T4	1,369 (31.4)	421 (45.5)	211 (41.6)	122 (31.9)	
N stage					<0.0001
N0	1,400 (32.1)	230 (24.8)	134 (26.4)	111 (29.0)	
N1	922 (21.2)	174 (18.8)	95 (18.7)	71 (18.5)	
N2	1,825 (41.9)	441 (47.6)	238 (46.9)	183 (47.8)	
N3	209 (4.8)	81 (8.8)	40 (7.9)	18 (4.7)	
M stage					0.0023
M0	4,030 (92.5)	825 (89.1)	458 (90.3)	357 (93.2)	
M1	326 (7.5)	101 (10.9)	49 (9.7)	26 (6.8)	
Stage					<0.0001
I	234 (5.4)	27 (2.9)	12 (2.4)	16 (4.2)	
II	511 (11.7)	93 (10.0)	56 (11.1)	42 (11.0)	
III	855 (19.6)	123 (13.3)	75 (14.8)	59 (15.4)	
IV	2,756 (63.3)	683 (73.8)	364 (71.8)	266 (69.5)	
Grade					0.0840
I	187 (4.3)	36 (3.9)	26 (5.1)	13 (3.4)	
II	1,761 (40.4)	426 (46.0)	208 (41.0)	146 (38.1)	
III/IV	1,372 (31.5)	261 (28.2)	164 (32.4)	122 (31.9)	
Unknown	1,036 (23.8)	203 (21.9)	109 (21.5)	102 (26.6)	
Dissected lymph node number					0.0035
0	2,186 (64.5)	519 (69.8)	293 (67.8)	226 (59.8)	
1–10	169 (5.0)	29 (3.9)	21 (4.9)	17 (4.5)	
11–20	108 (3.2)	12 (1.6)	6 (1.4)	8 (2.1)	
>20	7.2 (10.6)	59 (7.9)	42 (9.7)	38 (10.1)	
Unknown	571 (16.8)	125 (16.8)	70 (16.2)	89 (23.5)	
Surgery					<0.0001
No	3,416 (78.4)	791 (85.4)	416 (82.1)	301 (78.6)	
Yes	940 (21.6)	135 (14.6)	91 (18.0)	82 (21.4)	
Chemotherapy					0.2235
No	1,618 (37.1)	352 (38.0)	186 (36.7)	123 (32.1)	
Yes	2,738 (62.9)	574 (62.0)	321 (63.3)	260 (67.9)	
Radiotherapy					0.5664
No	981 (22.5)	219 (23.7)	117 (23.1)	77 (20.1)	
Yes	3,375 (77.5)	707 (76.4)	390 (76.9)	306 (79.9)	
Cancer specific death					0.0004
No	2,064 (47.4)	374 (40.4)	240 (47.3)	195 (50.9)	
Yes	2,292 (52.6)	552 (59.6)	267 (52.7)	188 (49.1)	
Overall death					<0.0001
No	1,126 (25.9)	179 (19.3)	154 (30.4)	121 (31.6)	
Yes	3,230 (74.2)	747 (80.7)	353 (69.6)	262 (68.4)	

API, Asian or Pacific Islander; NHB, non-Hispanic black; NHW, non-Hispanic white; SCC, squamous cell carcinoma.

^#^ Includes divorces, separated, widowed and unmarried.

### Cancer specific and overall survival rates in hypopharyngeal squamous carcinoma patients

**Fig 2:**
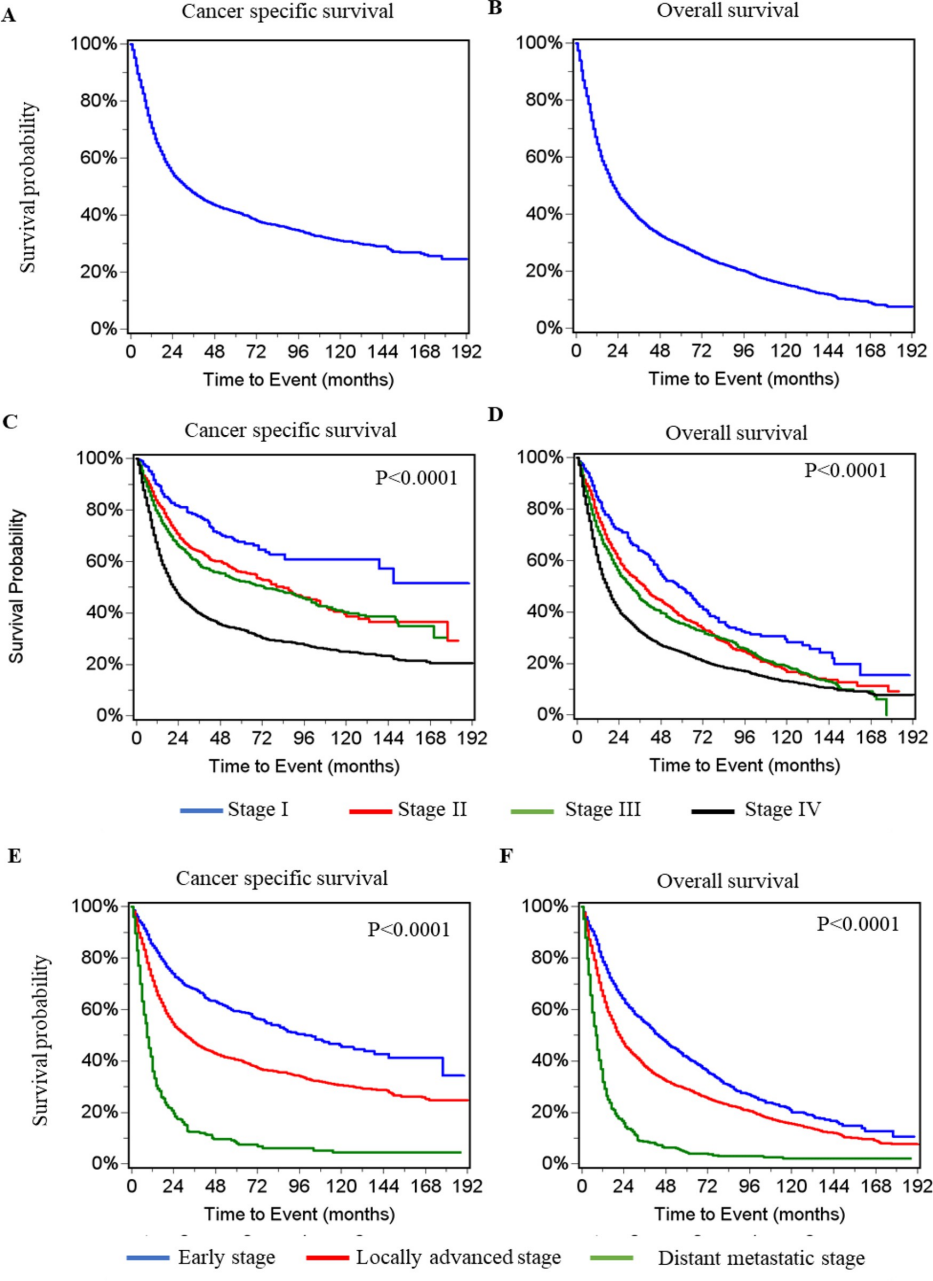
Kaplan–Meier survival curves in patients with hypopharyngeal squamous cell carcinoma (HPSCC). Cancer specific survival curves in all patients (**A**), in individual stages (**C**) and in early (I/II), locally advanced (III-IVb) and distant metastatic (IVc) stages (**E**). Overall survival curves in all patients (**B**), in individual stages (**D**) and in early (I/II), locally advanced (III-IVb) and distant metastatic (IVc) stages (**F**).

Both cancer specific and overall survival curves were constructed for all patients with HPSCC (Fig 2A and 2B). The 3- and 5-year cancer specific survival rates were 47.6% ± 0.7% and 41.2% ± 0.7%, respectively, and overall survival rates were 38.0% ± 0.6% and 28.9% ± 0.6%, respectively (Table 4). Survival curves for patients at individual stages were also constructed (Fig 2C and 2D). Patients at stage II demonstrated slightly better survivals than those at stage III. As expected, patients at an early stage (I and II) had better cancer specific and overall survival than those at locally advanced (III-IVb) or the distant metastatic stages (IVc) (Fig 2E and 2F). Their 3- and 5- year survival rates are presented at Table 4.

**Table 4:** Three- and five- year survival rates in patients with hypopharyngeal squamous cell carcinoma diagnosed during 2004–2019.

Viable (n)	Cancer specific survival (%)		Overall survival (%)	
	3-year	5-year	3-year	5-year
All patients (6,172)	47.6 ± 0.7	41.2 ± 0.7	38 ± 0.6	28.9 ± 0.6
Stage				
I (289)	77.1 ± 2.6	67.8 ± 3.1	63.7 ± 2.9	48.9 ± 3.1
II (702)	63.9 ± 2.0	56 ± 2.2	50.8 ± 2.0	37.6 ± 2.0
III (1,112)	58.1 ± 1.6	52.6 ± 1.7	44.6 ± 1.6	35.4 ± 1.5
IV (4,069)	39.6 ± 0.8	33.6 ± 0.9	32 ± 0.8	24.2 ± 0.7
Early (I/II) (991)	67.9 ± 1.6	59.5 ± 1.8	54.6 ± 1.6	41.0 ± 1.7
Locally advanced (III-IVb) (4,679)	46.7 ± 0.8	40.6 ± 0.8	37.5 ± 0.7	29.0 ± 0.7
Distant metastatic (IVc) (502)	12.6 ± 1.8	7.7 ± 1.6	8.7 ± 1.4	4.1 ± 1.0

### Racial disparity in cancer specific and overall survival in all patients with HPSCC

Survival curves for all HPSCC patients showed that NHB had significantly worst cancer specific and overall survivals among all races (S1A and S1B Fig). Multivariate survival analysis indicated that NHB patients had a significantly increased risk of overall death (HR = 1.11, 95% CI: 1.02–1.21, P = 0.0117), but not cancer specific death (HR = 1.07, 95% CI: 0.97–1.18, P = 0.1664). Hispanic patients were associated with better cancer specific survival HR = 0.85, 95% CI: 0.75–0.97, P = 0.0139) and overall survival (HR = 0.85, 95% CI: 0.76–0.95, P = 0.0041). API and NHW had a comparable prognosis. Age, marital status, year of diagnosis, income, tumor site, number of lymph node dissected, grade, T, N and M stages, chemotherapy, radiotherapy and surgery were significantly associated with survival in patients with HPSCC (S1 Table).

### Racial disparity in prognosis among HPSCC patients diagnosed at different stages

**Fig 3:**
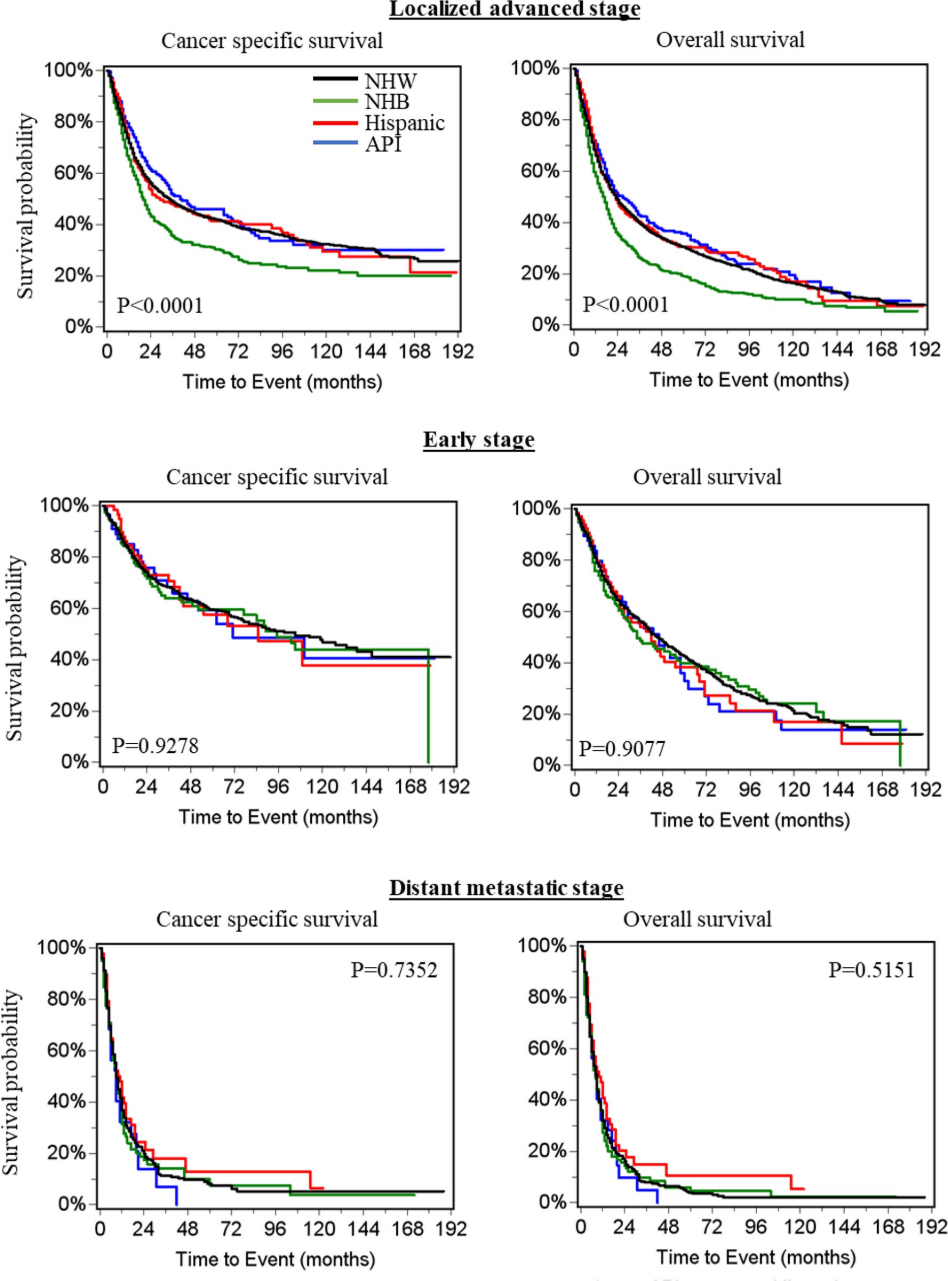
Kaplan–Meier survival curves for patients with stage T1 colorectal cancer in patients with hypopharyngeal squamous cell carcinoma (HPSCC) by stage and race. API, Asian or Pacific Islander; NHB, non-Hispanic black; NHW, non-Hispanic white.

In patients with HPSCC at the locally advanced stage, survival curves showed that NHB had significantly worse cancer specific and overall survival than other races (Fig 3A and 3B). There was no significant difference in cancer specific and overall survival among different races at Stage I/II (Fig 3C and 3D), and metastatic stage (Fig 3E and 3F).

Multivariate survival analysis showed that compared with NHW, NHB patients had a significant 13% increase in the risk of cancer specific death (HR = 1.13, 95% confidence interval (CI): 1.01–1.26, P = 0.0339) and 18% increase in the risk of overall death (HR = 1.18, 95% CI: 1.08–1.30, P = 0.0005). Hispanic patients were associated with better cancer specific survival (HR = 0.84, 95% CI: 0.73–0.98, P = 0.0239) and overall survival (HR = 0.84, 95% CI: 0.74–0.96, P = 0.0091) (Table 5). There was no significant difference in prognosis between NHW and API. Age, marital status, income, tumor site, tumor size, years of diagnosis, number of lymph nodes dissected, T, N, and M stages, grade, chemotherapy, radiotherapy and surgery were significantly associated with cancer specific and overall survivals in patients with HPSCC at the locally advanced stage (Table 5).

**Table 5:** Multivariate survival analysis of factors associated with locally advanced hypopharyngeal cancer.

Variable	Cancer Specific survival		Overall survival	
	HR (95% CI)	P value	HR (95% CI)	P value
Race				
NHW	1		1	
NHB	1.13 (1.01–1.26)	0.0339	1.18 (1.08–1.3)	0.0005
Hispanic	0.84 (0.73–0.98)	0.0239	0.84 (0.74–0.96)	0.0091
API	0.97 (0.82–1.16)	0.7610	0.97 (0.83–1.12)	0.6538
Age (years)				
≤ 65	1		1	
> 65	1.39 (1.28–1.51)	< .0001	1.5 (1.4–1.61)	< .0001
Marital status				
Married	1		1	
Unmarried	1.37 (1.26–1.49)	< .0001	1.36 (1.27–1.46)	< .0001
Unknown	1.14 (0.93–1.4)	0.202	1.06 (0.88–1.26)	0.5606
Year of diagnosis				
2004–2009	1		1	
2010–2014	0.87 (0.79–0.95)	0.0023	0.91 (0.84–0.98)	0.0167
2015–2019	0.77 (0.69–0.85)	< .0001	0.83 (0.75–0.91)	< .0001
Site				
Pyriform sinus	1		1	
Postcricoid region	1.22 (0.98–1.53)	0.0785	1.06 (0.86–1.3)	0.5917
Aryepiglottic fold	0.78 (0.64–0.95)	0.0123	0.82 (0.7–0.97)	0.0166
Posterior wall	1.29 (1.10–1.52)	0.0016	1.23 (1.08–1.41)	0.0028
Overlapping lesion	1.04 (0.84–1.28)	0.7328	1.01 (0.84–1.21)	0.9112
Hypopharynx, NOS	1.09 (0.99–1.19)	0.0684	1.06 (0.98–1.15)	0.1228
Annual household income ($)				
≤ 50000				
50001–75000	0.99 (0.89–1.10)	0.8018	0.97 (0.88–1.06)	0.4355
>75000	0.87 (0.78–0.99)	0.0281	0.88 (0.79–0.97)	0.0108
Size (cm)				
≤ 2	1		1	
2.1–5	1.08 (0.92–1.28)	0.3383	1.08 (0.95–1.24)	0.2451
>5	1.43 (1.19–1.73)	0.0002	1.35 (1.15–1.59)	0.0002
Unknown	1.26 (1.06–1.49)	0.0075	1.21 (1.05–1.39)	0.0078
T stage				
T1	1.00 (0.8–1.24)	0.9622	0.90 (0.75–1.08)	0.2668
T2	1		1	
T3	1.39 (1.24–1.57)	<0.0001	1.28 (1.16–1.42)	<0.0001
T4	1.78 (1.6–1.99)	<0.0001	1.50 (1.37–1.65)	<0.0001
N stage				
N0	1		1	
N1	1.33 (1.16–1.52)	< .0001	1.35 (1.20–1.51)	<0.0001
N2	1.71 (1.52–1.93)	<0.0001	1.55 (1.40–1.72)	<0.0001
N3	2.85 (2.37–3.43)	<0.0001	2.47 (2.10–2.91)	<0.0001
Grade				
I	1.04 (0.85–1.27)	0.684	1.01 (0.85–1.21)	0.8799
II	1		1	
III/IV	0.90 (0.82–0.99)	0.0272	0.90 (0.83–0.97)	0.009
Unknown	0.82 (0.74–0.91)	0.0003	0.84 (0.76–0.92)	0.0001
Dissected lymph node number				
0	1		1	
1–10	0.81 (0.68–0.97)	0.0253	0.78 (0.67–0.91)	0.0018
11–20	0.60 (0.45–0.8)	0.0004	0.65 (0.52–0.81)	0.0002
>20	0.69 (0.57–0.83)	<0.0001	0.62 (0.53–0.73)	<0.0001
Unknown	0.85 (0.76–0.96)	0.0070	0.88 (0.79–0.96)	0.0068
Surgery				
No	1		1	
Yes	0.69 (0.59–0.8)	<0.0001	0.74 (0.65–0.84)	<0.0001
Chemotherapy				
No	1		1	
Yes	0.63 (0.57–0.69)	<0.0001	0.61 (0.56–0.67)	<0.0001
Radiotherapy				
No	1		1	
Yes	0.40 (0.36–0.45)	<0.0001	0.43 (0.39–0.47)	<0.0001

API, Asian or Pacific Islander; NHB, non-Hispanic black; NHW, non-Hispanic white; SCC, squamous cell carcinoma.

^#^ Includes divorces, separated, widowed and unmarried.

There was no significant differences in cancer specific and overall survival among NHW, NHB, Hispanic and API patients with HPSCC at the early stage (Table 6). Age, marital status, T stage, surgery and radiotherapy were independent prognostic factors for patients. Chemotherapy was not associated with prognosis in patients with HPSCC at the early stage.

**Table 6:** Multivariate analysis of factors associated with survival in patients with early stage hypopharyngeal squamous cell carcinoma.

Variable	Cancer Specific survival		Overall survival	
	HR (95% CI)	P value	HR (95% CI)	P value
Race				
NHW	1		1	
NHB	0.97 (0.71–1.33)	0.8664	0.99 (0.78–1.26)	0.9330
Hispanic	0.93 (0.61–1.41)	0.7288	1.03 (0.76–1.41)	0.8367
API	1.06 (0.68–1.66)	0.7893	1.13 (0.81–1.58)	0.4744
Age (years)				
≤ 65	1		1	
> 65	1.26 (1.02–1.56)	0.0298	1.61 (1.37–1.89)	< .0001
Marital status				
Married	1		1	
Unmarried	1.51 (1.23–1.87)	0.0001	1.48 (1.27–1.74)	< .0001
Unknown	1.05 (0.57–1.94)	0.8689	0.92 (0.55–1.52)	0.7381
Annual house income ($)				
≤ 50000			1	
50001–75000			0.80 (0.65–0.98)	0.0327
>75000			0.82 (0.65–1.05)	0.1125
T stage				
T1	1		1	
T2	1.47 (1.15–1.87)	0.0023	1.24 (1.04–1.47)	0.0190
Surgery				
No	1		1	
Yes	0.46 (0.35–0.6)	<0.0001	0.50 (0.41–0.61)	<0.0001
Radiotherapy				
No	1		1	
Yes	0.51 (0.4–0.65)	<0.0001	0.57 (0.47–0.68)	<0.0001

AIAN, American Indian and Alaska Native; API, Asian or Pacific Islander; NHB, non-Hispanic black; NHW, non-Hispanic white.

^#^ Includes divorces, separated, widowed and unmarried.

In patients with HPSCC at the distant metastatic stage, API and NHW had no significant difference in both cancer specific and overall survival. In contrast, Hispanic patients had significantly better overall survival (HR = 0.69, 95% CI: 0.50–0.95, P = 0.0248) than NHW with HPSCC at the distant metastatic stage, but no significant difference in cancer specific survival (Table 7). Our study also showed that T, N, and M stages, all treatments including surgery, and radiotherapy were associated with cancer specific and overall survival in patients.

**Table 7:** Multivariate analysis of factors associated survival in patients with distant metastatic hypopharyngeal squamous cell carcinoma.

Variable	Cancer Specific survival		Overall survival	
	HR (95% CI)	P value	HR (95% CI)	P value
Race				
NHW	1		1	
NHB	0.93 (0.72–1.21)	0.5947	0.92 (0.60–1.40)	0.6902
Hispanic	0.75 (0.54–1.06)	0.0988	0.69 (0.50–0.95)	0.0248
API	0.99 (0.64–1.53)	0.9546	0.92 (0.72–1.17)	0.4888
T stage				
T1	0.49 (0.27–0.89)	0.0193	0.51 (0.30–0.87)	0.0133
T2	1.10 (0.82–1.48)	0.5299	1.17 (0.90–1.54)	0.2487
T3	1		1	
T4	1.04 (0.79–1.37)	0.7751	0.97 (0.75–1.25)	0.8019
N stage				
N0	1		1	
N1	1.55 (1.07–2.25)	0.0214	1.6 (1.13–2.26)	0.0077
N2	1.58 (1.13–2.2)	0.0072	1.59 (1.17–2.17)	0.0031
N3	1.98 (1.30–3.02)	0.0015	1.99 (1.34–2.96)	0.0007
Surgery				
No	1		1	
Yes	0.59 (0.39–0.89)	0.0121	0.61 (0.42–0.89)	0.0102
Chemotherapy				
No	1		1	
Yes	0.36 (0.28–0.44)	< .0001	0.35 (0.28–0.43)	< .0001
Radiotherapy				
No	1		1	
Yes	0.62 (0.50–0.76)	< .0001	0.63 (0.52–0.77)	< .0001

API, Asian or Pacific Islander; NHB, non-Hispanic black; NHW, non-Hispanic white.

## Discussions

This study began by first assessing racial disparity in HPSCC incidence and its temporal change during 2004–2019 using a database covering the entire population of the US. Our results revealed that males had a much higher overall AAIR of HPSCC than females. Among all races, NHB had had the highest AAIRs in males and females during the entire study period and between 2017 and 2019. Male NHW, NHB and Hispanic as well as female NHW and NHB had decreased AAIR of HPSCC, while NHB male had the fastest rate of decrease. Previous studies reported that the continuous decrease of overall HPSCC incidence in all races/ethnicities during 1973–2010 [1, 4]. Together, these findings suggest that the HPSCC incidence has been continuously decreasing in the past 4 decades.

The declining incidence of HPSCC is mainly due to the decreased prevalence of smoking in recent decades [22, 23]. Smoking is known as an important risk factor for hypopharyngeal and many other cancers. Like HPSCC, other smoking related cancers display reduced incidences in recent decades [24, 25]. Men have a higher smoking prevalence which is also related to a higher incidence of HPSCC in men compared with women. The decline in the incidence of HPSCC may be associated with reduced human papillomavirus (HPV) infection by vaccine use in the last decade [26]. Alcohol consumption and smoking concomitantly increase the risk of cancer in HPSCC and other head and neck cancers [27]. However, alcohol consumption has steadily increased in recent decades [28, 29]. It warrants further studies to explore underlying causes for the decreased trend of HPSCC incidence and racial disparity. On the other hand, this and other studies indicated the majority of HPSCC was diagnosed at the advanced stages [30, 31], implying that the decreased incidence of HPSCC is not caused by the increased early screening and detection. There is a great need to develop more effective ways of early detection for HPSCC.

We further determined the racial disparity of clinicopathological features in a total of 6,172 HPSCC patients from the SEER 17 database between 2004 and 2019. Our result revealed that NHB bore an unequally heavy burden of HPSCC. In addition to higher incidence of HPSCC, NHB had the highest proportion of the stage IV with the largest sized tumors as compared to other races. Similar to the finding from a previous study [32], NHB with HPSCC were more likely to be diagnosed at younger ages; however, they were the least likely to receive surgery. Similarly, NHB with head and neck cancers were previously reported to be more likely to refuse surgery and select nonsurgical treatment [33–35]. Our results showed no difference in the proportion of patients receiving chemotherapy or radiotherapy between NHB and other races. NHB had higher proportions of deaths due to cancer or all causes. Multivariate survival analysis showed that compared with NHW, NHB had significantly higher risks of cancer specific and overall mortality in HPSCC at the locally advanced stage, although not at the early stage (I/II), or the distant metastatic stage. Socioeconomic factors, such as income, education level, employment and residence place, may have a significant impact on stage at diagnosis, treatment, and survival [36]. The heavier burden of HPSCC in NHB is likely due to their limited access to health care and low socioeconomic status [37–39]. Indeed, NHB patients in this study were shown to have relatively low socioeconomic status and income, be from the South (60.4%) and have poor health care quality.

Similar to our findings, several recent studies have reported a worse prognosis of HPSCC in NHB compared with NHW. Lin et al. conducted a similar study in patients (n = 2007) with HPSCC from the SEER database during 2004–2015. Black patients were shown to have increased risk of cancer-specific death than whites or other races [10]. A SEER based study obtained a total of 864 patients with locally advanced HPSCC during 2010–2015. Black patients were shown to have significantly increased risk of overall death, but not cancer specific death, than whites or other races [11]. Tian et al. obtained 2198 HPSCC patients from the SEER database between 2010 and 2016. Survival analysis results revealed that being black was associated with a worse overall survival compared with whites, but there was no significant difference in overall survival between White or other races [12]. Another SEER based study obtained 2021 HPSCC patients diagnosed between 2010 and 2015. Their results revealed that black patients had a significantly worse cancer specific death compared to whites and other races in combination [13]. Using the National Cancer Database (NCDB), prognostic factors were determined in a total of 6,055 adult patients with locally advanced HPSCC diagnosed between 2004 and 2015. NHB had a significantly increased risk of overall death compared to NHW [17]. Chiruvella et al. found that black patients had the highest incidence-based mortality rates for oropharyngeal cancer and HPSCC compared to whites or other races from 2000 to 2017 [40].

Conversely, several other studies found no prognostic difference in HPSCC between NHB and NHW or other races. From 2010 to 2015, 857 patients with HPSCC (T2‐T4aM0) treated with surgery or definitive chemoradiotherapy were retrieved from the SEER database. Multivariate survival analyses were performed in T2‐3 and T4a subgroups. Their result revealed that black patients or other races demonstrated no significant difference in overall survival compared to white patients [15]. Another SEER based study enrolled 1144 HPSCC patients treated with surgery between 2004 and 2015. No significant difference in cancer specific survival was observed between whites and blacks, or between white and other races [14]. Using the SEER database, another study recruited 3,172 HPSCC patients aged 60 and above years old diagnosed from 2004 to 2018. Multivariate survival analysis indicated that there was no significant difference in cancer specific survival between white and black patients [16]. Kuo et al. identified a total of 3,357 adult patients with primary HPSCC without distant metastases from the NCDB between 1998 and 2011. There was no significant difference in overall survival in other races compared to NHW [41]. A negative racial disparity in overall survival was also reported between black and white, or between other and White [4], or between African American and Caucasian American [31]. S2 Table summarizes of these previous studies on racial disparities in the prognosis of patients with HPSCC.

Several reasons may explain the inconsistent results of racial disparity in the prognosis of HPSCC patients. Some studies only included subgroups of HPSCC patients, for example age 60 and above, or those treated by surgery, or with locally advanced stages. Our data showed that prognostic factors changed among patients within different disease stages. Various follow-up times were adopted in different studies. Many studies separated patients into training and validation groups for prognosis predicting models. Consequently, case numbers and the power to discern the race disparity were reduced in the multivariate survival analysis. This study revealed that Hispanic patients may have a better prognosis than NHW. Many previous studies didn’t separate Hispanics from white and black patients, which may confound the result of the racial disparity.

The majority (79.9%) of Hispanic patients in this study were from the Western region. They had a greater proportion of higher median household income (> $75,000) than NHW and NHB. A recent CDC report found significantly lower tobacco use in Hispanics compared to NHW or NHB [22]. Hispanics had significantly better cancer specific and overall survival than NHW when analyzing all HPSCC patients, or in locally advanced patients, and better overall survival in the distant metastatic patients. There was no significant difference in prognosis among all races when only analyzing stage I/II patients. Better prognosis in Hispanic patients has been reported in other head and neck cancers [7, 42]. The trend of a better prognosis and other health outcomes in the Hispanic population contrasts to their general low socioeconomic status. This has been previously described as the Hispanic paradox [43]. Hypothesized multi-factorial causes, such as younger population, missing deaths, and/or return migration, have been implicated in this paradox [44–46]. Other studies reported no significant difference in overall survival between Hispanic and non-Hispanics [17, 41, 47, 48].

Based on N stage, 69.6% HPSCC patients were diagnosed with lymph node metastasis. N stage was shown as an independent factor correlated with a poorer prognosis in patients with HPSCC in this study and many previous studies [10, 11, 13, 14, 17, 49, 50]. Positive lymph node number or its ratio to total number of dissected lymph nodes were correlated with a prognosis of patients with HPSCC in multiple studies [51–54]. Survival analyses of our data showed that the number of lymph nodes dissected was a prognostic factor for HPSCC patients. Similarly, removal and identification of 18 or more lymph nodes was associated with improved overall survival and lower rates of local-regional failure in head and neck squamous cell carcinoma [55]. Since HPSCC has a high incidence of lymph node metastasis, better management of lymph nodes may help improve the diagnosis, treatment and survival.

Multivariate survival analysis also showed that patients diagnosed in more recent years had a better prognosis than those diagnosed in older years. Patients from this study had a better 5-year cancer specific survival rate (41.2% vs 33.4%) compared to those reported previously [3]. These findings support the improved therapeutic efficacy for HPSCC in recent years. Moreover, surgery, chemotherapy and radiotherapy were associated with significantly improved overall and specific survivals compared with untreated counterparts in all patients or in different stage groups, except that chemotherapy was not associated with the prognosis in early stage I/II patients. Notably, a much higher proportion of patients were receiving chemotherapy (63.1%) and radiotherapy (77.4%) than surgery (20.2%). Combined chemotherapy and radiotherapy are considered alternative options of laryngeal preservation surgery [56, 57]. More studies are needed to determine optimal combined treatments to improve both survival and quality of life in these patients.

This SEER based study has its limitations. Information of many other possible HPSCC related risk factors, such as smoking, HPV infection and alcohol consumption, are not available, which limits the capability to explore these as plausible causes for decreased incidence and racial disparity. Patients’ comorbidities affect the choice of treatments and overall survival. This set of data is not available in the database. It is inevitable that there is selection bias in this retrospective study in which patients were not randomly assigned to the treatment groups. There was no detailed information of treatments, such as duration or dose of radiotherapy and chemotherapy. In addition, the database lacks the data of disease recurrences and quality of life. These outcomes are valuable for evaluation of racial disparities. The strength of our study is that we have utilized the database covering the entire nation, to determine HPSCC incidence and trends, and have included thus far, the largest number of HPSCC cancer patients to evaluate racial disparities in clinicopathological characteristics and prognosis.

## Conclusions

Using the database covering the entire nation, this study reveals that HPSCC incidence displays continuous decreases and racial disparity. The majority of the disease is diagnosed at the advanced stage. NHB had the highest burden of HPSCC with the worst prognosis. More studies are needed to curtail racial disparities and improve early detection.

## Supporting information

S1 Fig Kaplan–Meier survival curves for patients with hypopharyngeal squamous cell carcinoma by race. (**A**) Cancer specific survival. (**B**) Overall survival. API, Asian or Pacific Islander; NHB, non-Hispanic black; NHW, non-Hispanic white.
(PDF)

S1 Table Multivariate analysis of factors associated with survival in all patients with hypopharyngeal squamous cell carcinoma. (PDF)

S2 Table Summary of previous studies on racial disparities in the prognosis of patients with hypopharyngeal squamous cell carcinoma. (PDF)
