# Supplementary material for: Updated racial disparities in incidence, clinicopathological features and prognosis of hypopharyngeal squamous carcinoma in the United States
Source: PLoS One. 2023 Mar 16;18(3):e0282603. doi: 10.1371/journal.pone.0282603 (PMC10019746; doi:10.1371/journal.pone.0282603)

Supporting information

S1 Fig. Kaplan–Meier survival curves for patients with hypopharyngeal squamous cell carcinoma by race. (**A**) Cancer specific survival. (**B**) Overall survival. API, Asian or Pacific Islander; NHB, non-Hispanic black; NHW, non-Hispanic white.

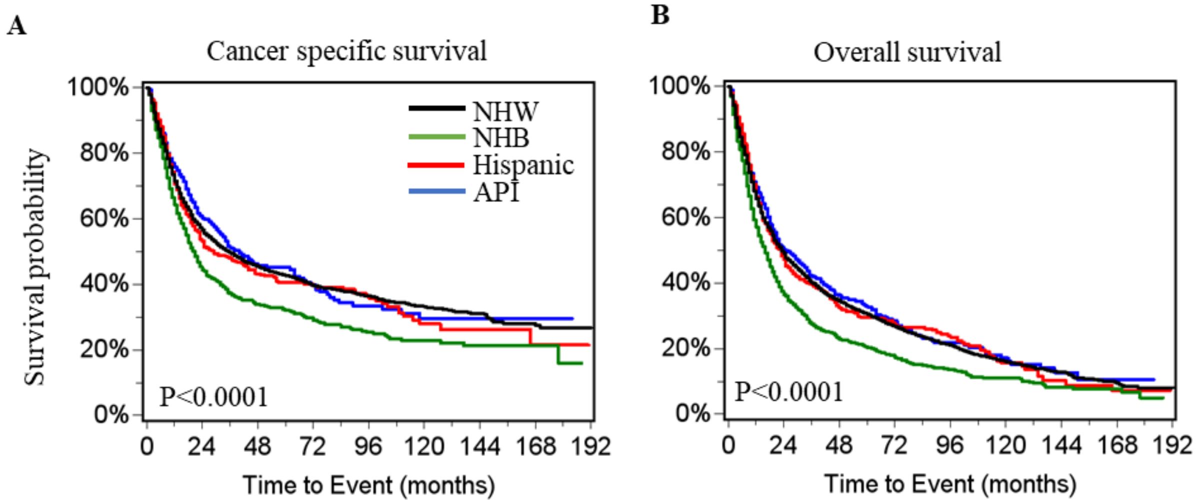

Supplement: S1 Fig — (A) Cancer specific survival. (B) Overall survival. API, Asian or Pacific Islander; NHB, non-Hispanic black; NHW, non-Hispanic white. (PDF) [file pone.0282603.s001.pdf]
