# Supplementary material for: Updated racial disparities in incidence, clinicopathological features and prognosis of hypopharyngeal squamous carcinoma in the United States
Source: PLoS One. 2023 Mar 16;18(3):e0282603. doi: 10.1371/journal.pone.0282603 (PMC10019746; doi:10.1371/journal.pone.0282603)
Supplement: S1 Table — (PDF) [file pone.0282603.s002.pdf]

Supporting information
S1 Table.
Multivariate analysis of factors associated with survival in all patients with hypopharyngeal squamous cell carcinoma.

| Variable | Cancer Specific survival | | Overall survival | |
|---|---|---|---|---|
| | HR (95% CI) | P value | HR (95% CI) | P value |
| Race | | | | |
| NHW | 1 | | 1 | |
| NHB | 1.07 (0.97-1.18) | 0.1664 | 1.11 (1.02-1.21) | 0.0117 |
| Hispanic | 0.85 (0.75-0.97) | 0.0139 | 0.85 (0.76-0.95) | 0.0041 |
| API | 1.00 (0.86-1.17) | 0.9899 | 1.00 (0.88-1.14) | 0.9929 |
| Age (years) | | | | |
| ≤ 65 | 1 | | 1 | |
| > 65 | 1.32 (1.23-1.42) | <0.0001 | 1.45 (1.37-1.55) | <0.0001 |
| Marital status | | | | |
| Married | 1 | | 1 | |
| Unmarried | 1.36 (1.26-1.46) | <0.0001 | 1.36 (1.28-1.44) | <0.0001 |
| Unknown | 1.16 (0.96-1.39) | 0.122 | 1.07 (0.91-1.25) | 0.4468 |
| Year of diagnosis | | | | |
| 2004-2009 | 1 | | 1 | |
| 2010-2014 | 0.87 (0.80-0.94) | 0.0005 | 0.90 (0.84-0.96) | 0.0018 |
| 2015-2019 | 0.82 (0.74-0.9) | <0.0001 | 0.85 (0.78-0.92) | <0.0001 |
| Annual household income ($) | | | | |
| ≤ 50000 | 1 | | 1 | |
| 50001-75000 | 0.99 (0.90-1.09) | 0.8535 | 0.95 (0.88-1.03) | 0.2008 |
| >75000 | 0.88 (0.79-0.98) | 0.0151 | 0.88 (0.80-0.96) | 0.0034 |
| Region | | | | |
| West | 1 | | | |
| South | 1.00 (0.84-1.19) | 0.9784 | | |
| Midwest | 0.92 (0.85-1.00) | 0.0486 | | |
| Northeast | 0.84 (0.77-0.92) | 0.0002 | | |
| Site | | | | |
| Pyriform sinus | 1 | | 1 | |
| Postcricoid region | 1.16 (0.95-1.41) | 0.1483 | 1.07 (0.9-1.27) | 0.4787 |
| Aryepiglottic fold | 0.77 (0.66-0.91) | 0.0016 | 0.83 (0.73-0.94) | 0.0042 |
| Posterior wall | 1.21 (1.05-1.39) | 0.0071 | 1.18 (1.05-1.33) | 0.0046 |
| Overlapping lesion | 0.94 (0.78-1.13) | 0.5096 | 0.96 (0.82-1.12) | 0.5872 |
| Hypopharynx, NOS | 1.06 (0.98-1.15) | 0.1767 | 1.03 (0.96-1.1) | 0.3790 |
| Size (cm) | | | | |
| ≤ 2 | 1 | | 1 | |
| 2.1-5 | 1.04 (0.91-1.19) | 0.5630 | 1.08 (0.96-1.21) | 0.1916 |
| >5 | 1.35 (1.15-1.58) | 0.0003 | 1.33 (1.16-1.53) | <0.0001 |
| Unknown | 1.24 (1.07-1.42) | 0.0032 | 1.23 (1.10-1.39) | 0.0005 |
| T stage | | | | |

| | | | | |
|---|---|---|---|---|
| T1 | 0.56 (0.47-0.68) | <0.0001 | 0.63 (0.54-0.74) | <0.0001 |
| T2 | 0.73 (0.66-0.81) | <0.0001 | 0.79 (0.73-0.86) | <0.0001 |
| T3 | 1 | | 1 | |
| T4 | 1.25 (1.14-1.38) | <0.0001 | 1.16 (1.07-1.26) | 0.0003 |
| N stage | | | | |
| N0 | 1 | | 1 | |
| N1 | 1.34 (1.21-1.5) | <0.0001 | 1.33 (1.22-1.46) | <0.0001 |
| N2 | 1.68 (1.52-1.84) | <0.0001 | 1.51 (1.4-1.64) | <0.0001 |
| N3 | 2.55 (2.18-2.98) | <0.0001 | 2.24 (1.95-2.58) | <0.0001 |
| M stage | | | | |
| M0 | 1 | | 1 | |
| M1 | 1.97 (1.76-2.2) | <0.0001 | 1.85 (1.67-2.05) | <0.0001 |
| Number of lymph node harvested | | | | |
| 0 | 1 | | 1 | |
| 1-10 | 0.90 (0.76-1.06) | 0.1943 | 0.86 (0.75-0.99) | 0.0336 |
| 11-20 | 0.71 (0.55-0.91) | 0.0071 | 0.77 (0.63-0.94) | 0.0104 |
| > 20 | 0.79 (0.67-0.93) | 0.0046 | 0.73 (0.64-0.84) | <0.0001 |
| Unknown | 0.93 (0.84-1.03) | 0.1877 | 0.92 (0.85-1.01) | 0.0786 |
| Grade | | | | |
| I | 1.00 (0.84-1.19) | 0.9784 | 0.98 (0.85-1.14) | 0.805 |
| II | 1 | | 1 | |
| III/IV | 0.92 (0.85-1) | 0.0486 | 0.93 (0.87-1) | 0.0368 |
| Unknown | 0.84 (0.77-0.92) | 0.0002 | 0.87 (0.8-0.94) | 0.0005 |
| Surgery | | | | |
| No | 1 | | 1 | |
| Yes | 0.61 (0.53-0.69) | <0.0001 | 0.64 (0.57-0.71) | <0.0001 |
| Chemotherapy | | | | |
| No | 1 | | 1 | |
| Yes | 0.62 (0.57-0.67) | <0.0001 | 0.61 (0.56-0.65) | <0.0001 |
| Radiotherapy | | | | |
| No | 1 | | 1 | |
| Yes | 0.46 (0.42-0.5) | <0.0001 | 0.49 (0.46-0.53) | <0.0001 |

API, Asian or Pacific Islander; NHB, non-Hispanic black; NHW, non-Hispanic white; SCC, squamous cell carcinoma.

[#] Includes divorces, separated, widowed and unmarried.
