# Supplementary material for: Updated racial disparities in incidence, clinicopathological features and prognosis of hypopharyngeal squamous carcinoma in the United States
Source: PLoS One. 2023 Mar 16;18(3):e0282603. doi: 10.1371/journal.pone.0282603 (PMC10019746; doi:10.1371/journal.pone.0282603)
Supplement: S2 Table — (PDF) [file pone.0282603.s003.pdf]

Supporting information

S2 Table.
Summary of previous studies on racial disparities in the prognosis of patients with hypopharyngeal squamous cell carcinoma.

Author [reference]	Study period	HPSCC patients (Source)	Number of patients	Race group	Survival outcome
Lin et al [1]	2004-2015	All stages (SEER)	Training cohort 1,006; Validation cohort 1,001	Black, white and other	Black had a worse cancer specific survival than white, or other races (no detailed data).
Yang et al [2]	2010-2015	Locally advanced (SEER)	Training set 608; Validation set 256	Black, white and other	White vs black (HR=0.612, P=0.001), other vs black (HR 0.617, P=0.046) for overall survival. No significant difference between white vs black or other vs black for cancer specific survival.
Tian et al [3]	2010-2016	All stages (SEER)	Training set 1758; Validation set 440	Black, white and other	Black vs white (HR=1.333 P=0.001). No difference between white and other for overall survival.
Tang et al [4]	2010-2015	All stages (SEER)	Training set 1415; Validation set 606	Black, white and other	Black vs white and other (HR=1.266, P=0.014) on cancer specific survival.
Hochfelder et al [5]	2004-2015	Stage III or IV, M0 (National Cancer Database)	6,055	NHW, NHB, Hispanic, non-Hispanic other	NHB vs NHW (HR=1.13, P=0.012); No difference between NHW and Hispanic, or between NHW and non Hispanic other for overall survival.
Chiruvella et al [6]	20102-2017	All stages (SEER)	No data.	Caucasian/white, African American/black, American Indian/Alaskan native and Asian/Pacific Islander	Incidence based mortality rates (per 1,000) in males and females, respectively, were Caucasian/white 7.3 and 1.7, African American/black 15.2 and 2.3, American Indian/Alaskan Native 6.6 and 0.7, Asian/Pacific Islander 4.9 and 0.7.
Wang et al [7]	2004-2015	Surgery treated patients (SEER)	Training set 688; Validation set 456	Black, white and other	No difference among races in cancer specific survival among races.
Kim et al [8]	2010-2015	T2-T4aM0 (SEER)	Surgery 209; Chemoradiotherapy 648	Black, white and other	No significant difference between black vs white, or other vs White in all patients, T2-3 patient, or T4a patients for overall survival.

Wang et al [9]	2004-2018	All stages (SEER)	Training set 2,219; Validation set 953	Black, white and other	No significant difference in cancer specific survival among races.
Kuo et al [10]	Diagnosed 2003-2006 followed up to 2011	Non-distant metastatic (M0) (National Cancer Data Base)	3357	non-Hispanic white, African/ African American, Hispanic, Asian/Pacific Islander, unknown/other	No significantly difference between all other races and non-Hispanic white in overall survival.
Kuo et al [11]	1988-2005	Non-distant metastatic (SEER)	2,334	Black, white and other	No significant difference between white and black or between white and other for overall survival.
Vengaloor Thomas et al [12]	1994-2018	All stages (The University of Mississippi Medical Center)	144	Caucasian American, African American, unknown/other	No difference between African American and Caucasian American for overall survival.

API, Asian or Pacific Islander; HPSCC: hypopharyngeal squamous cell carcinoma; HR, hazard ratio; NHB, non-Hispanic black; NHW, non-Hispanic white.

References:

1. Lin Z, Lin H, Lin C: **Dynamic prediction of cancer-specific survival for primary hypopharyngeal squamous cell carcinoma**. *Int J Clin Oncol* 2020, **25**(7):1260-1269.
2. Yang H, Zeng M, Cao S, Jin L: **Nomograms predicting prognosis for locally advanced hypopharyngeal squamous cell carcinoma**. *Eur Arch Otorhinolaryngol* 2022, **279**(6):3041-3052.
3. Tian S, Li Q, Li R, Chen X, Tao Z, Gong H, Wang X, Hu X: **Development and Validation of a Prognostic Nomogram for Hypopharyngeal Carcinoma**. *Front Oncol* 2021, **11**:696952.
4. Tang X, Pang T, Yan WF, Qian WL, Gong YL, Yang ZG: **A novel prognostic model predicting the long-term cancer-specific survival for patients with hypopharyngeal squamous cell carcinoma**. *BMC Cancer* 2020, **20**(1):1095.
5. Hochfelder CG, Mehta V, Kabarriti R, McGinn AP, Castellucci E, Ow TJ: **Survival analysis of patients with advanced hypopharyngeal cancer comparing patients who received primary surgery to those who received chemoradiation: An analysis of the NCDB**. *Oral Oncol* 2021, **121**:105470.
6. Chiruvella V, Guddati AK: **Analysis of Race and Gender Disparities in Mortality Trends from Patients Diagnosed with Nasopharyngeal, Oropharyngeal and Hypopharyngeal Cancer from 2000 to 2017**. *Int J Gen Med* 2021, **14**:6315-6323.
7. Wang K, Xu X, Xiao R, Du D, Wang L, Zhang H, Lv Z, Li X, Li G: **Development and validation of a nomogram to predict cancer-specific survival in patients with hypopharyngeal squamous cell carcinoma treated with primary surgery**. *J Int Med Res* 2021, **49**(12):3000605211067414.
8. Kim YJ, Lee R: **Surgery vs. radiotherapy for locally advanced hypopharyngeal cancer in the contemporary era: A population-based study**. *Cancer Med* 2018, **7**(12):5889-5900.
9. Wang J, Liu X, Tang J, Zhang Q, Zhao Y: **A Web-Based Prediction Model for Cancer-Specific Survival of Elderly Patients With Hypopharyngeal Squamous Cell Carcinomas: A Population-Based Study**. *Front Public Health* 2021, **9**:815631.

10. Kuo P, Sosa JA, Burtness BA, Husain ZA, Mehra S, Roman SA, Yarbrough WG, Judson BL: **Treatment trends and survival effects of chemotherapy for hypopharyngeal cancer: Analysis of the National Cancer Data Base**. *Cancer* 2016, **122**(12):1853-1860.
11. Kuo P, Chen MM, Decker RH, Yarbrough WG, Judson BL: **Hypopharyngeal cancer incidence, treatment, and survival: temporal trends in the United States**. *Laryngoscope* 2014, **124**(9):2064-2069.
12. Vengaloor Thomas T, Krishna K, Ahmed HZ, Mundra E, Abraham A, Bhanat E, Nittala MR, Packianathan S, Vijayakumar S: **A 25-year Experience at an Academic Medical Center in the United States: Are There Racial Disparities in the Prognosis of Patients Diagnosed With Hypopharyngeal Carcinoma?** *Cureus* 2020, **12**(11):e11306.
